# Twisting Tourniquet^©^ Technique: introducing *Schnogh*, a novel device and its effectiveness in treating primary and secondary lymphedema of extremities

**DOI:** 10.1002/cam4.498

**Published:** 2015-08-06

**Authors:** Narumon Chanwimalueang, Wichai Ekataksin, Parkpoom Piyaman, Gedsuda Pattanapen, Borimas K Hanboon

**Affiliations:** 2 Liver Research Unit, Faculty of Tropical Medicine, Mahidol UniversityBangkok, Thailand; 1 Lymphology Institute of Thailand and Lymphedema Day Care CenterNontaburi, Thailand; 3 Lymphedema Clinic, Tokushukai Overseas Medical Cooperation Center, Tateyama HospitalChiba, Japan

**Keywords:** Compression therapy, filariasis, lymphedema, Schnogh, twisting tourniquet, vegan

## Abstract

Twisting Tourniquet^©^ or in Thai “Schnogh” is a new invention for compression therapy of lymphedema. Twisting Tourniquet^©^ Technique (TTT) is totally noninvasive for lymphedema management. After the amazing successful evidence in the first series of 28 patients, we have conducted preliminary studies in lymphedema clinics. It was found that the combination of gradually increasing constriction force by Schnogh until desired pressure was reached and maintained for 15 min, followed by a 5-min release, doing repeatedly this compression-decompression for at least 10 sessions a day, can generate acceptable results. The aim of the study was to evaluate the scientific effectiveness and establish a treatment protocol of TTT proposed as a therapeutic approach for clinical management of lymphedema. During 2006–2013, from over 3500 patients, 647 with primary/secondary lymphedema passed inclusion criteria, 307 for upper, and 340 for lower extremity. In the 5-day course of TTT, each day patients underwent 10 sessions of a 15-min compression followed by a 5-min decompression. Vegan diet was encouraged as an adjuvant therapy. Among lymphedema patients whose spectrum of edema severity ranged from mild to gigantic, TTT yielded an average volume reduction rate (VR) at 50.2% and 55.6%, making the average edema reduction volume attained at 463 and 1856 mL for upper and lower limb, respectively. The uniformed practice by Schnogh which supports a continual compression–decompression maneuver over 3.5 h daily for five consecutive days could induce an average VR at above half of the swelling in extremities of 647 patients. Schnogh is therefore effective in clinical management of lymphedema under TTT treatment of fibroblastic interstitium.

## Introduction

At any single point of time, lymph is produced in largest quantity in liver 1. As much as 25–50% of lymphatic flow in thoracic duct is contributed from hepatic origin 2, whereas less than 10% is originated from limb and cervical trunks 3. Scientists have long wondered how tissue fluid in the portal tract migrates to the lymphatics 4–6. The mystery was solved by the discovery of honeycomb architecture of fibroblastic interstitium 7.

The quest why the liver, which generates so huge amount of lymph, itself so rarely suffers from lymphedema lies in its histoorganization with some hepatocytes dedicatedly embracing each portal tract as limiting plate 8. It is the limiting plate, which enwraps the portal tract, the lymph-enriched compartment, that prevents the tract from abrupt distending, and with increased tension in lobular architectonics under inflow load, limits the tract from over-expanding. This resilience nature, as a result, equips the organ with a unique characteristic of expansibility and retractability constituted within low-ranged pressure gradient 9.

We found from the microscopic points of view that lymphedema is a diffuse filling and distending of honeycomb interconnected microchambers. However, in the opposite way, lymphatic spaces that are distensible can be collapsible by the compression force and lymphatic pathways accordingly are subject to remodeling. Therefore, expansion should be decreased with compression force from some tools applied from outside to reduce the swelling of the extremities. With this principle in mind, in 2006, we attempted inventing simple tools for compressing around a swollen limb in order to progressively reduce the size of swollen extremity. Starting de novo from ordinary materials, a prototype Schnogh, or Twisting Tourniquet^©^, was designed, tested, modified, and continually improved into its current form.

Due to total lack of lymphedema management in Thailand so far, all patients have been left virtually untreated, or treated minimally, conservatively, or by some surgical intervention, essentially without proper care for years or decades, even throughout their lives. After the amazing success evident in the first 28 patients, we have conducted preliminary studies in which tens and hundreds of suffered lymphedema patients participated willingly.

This study aims to present a morphofunctional practicalness of Twisting Tourniquet^©^ Technique (TTT) and demonstrate its clinical effectiveness. We also aim to establish a treatment protocol of TTT proposed as a routine approach for clinical management of lymphedema.

## Materials, Patients, and Methods

### Physical design of Schnogh or Twisting Tourniquet^©^

The practice of our decongestive therapy owes its principal part to the maneuver of Schnogh, the Twisting Tourquet^©^, which, when applied appropriately around a limb, is a manually adjustable tightening device. Our first few hundred sets of Schnogh were crafted at a laboratory level from ordinary materials available in shops of fabric and sewing/embroidery stuff; mass production was later done by professional sewers. Although more than 10 models of Schnogh have been invented, we primarily use only two or three at the Lymphology Institute of Thailand in routine practice. They are the *Paired Schnogh*, which can be operated bimanually, and the *Cuffed Schnogh*, or its modification, the *Multi-belted Schnogh*, which can be operated even with one free hand. These three models are built not for trained personnel, but under a patient-oriented design with an intuitive ease of use, and are environmentally friendly, that is, a lifetime reuse with no piece to discard. A brief description of the device is given as follows.

**Figure 1:**
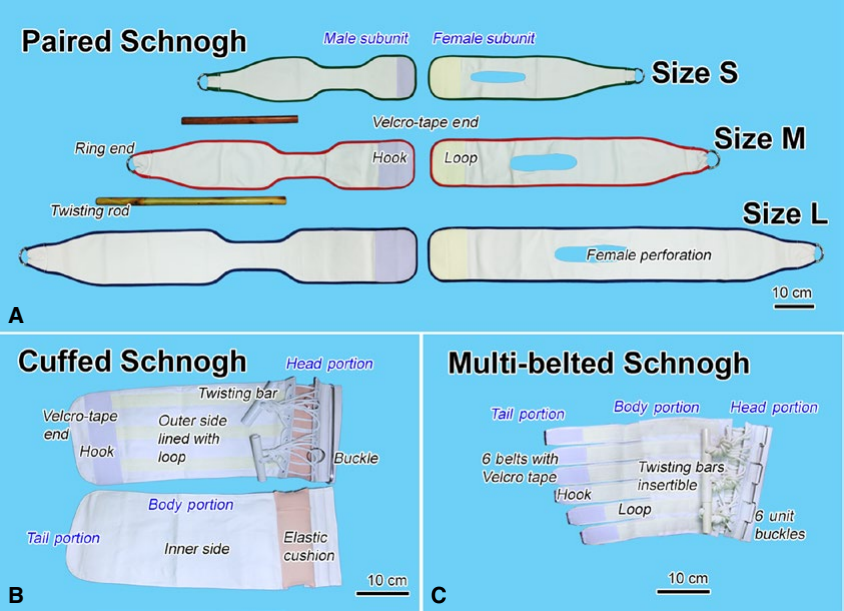
Three types of Schnogh or Twisting Tourquet©. (A) *Paired Schnogh*, each with a male and a female subunit for use with lower limbs. (B) *Cuffed Schnogh* for upper/lower limbs, shown on an outer and inner aspect, is consisted of a broad band body, having a expansible head portion with a series of rope work and a full-width metal buckle, and a tail portion with a Velcro-tape end. The ropes interlaced in a canvas shoe fashion, work with two twisting bars of different sizes, a smaller male bar insertable into a larger female bar. (C) *Multibelted Schnogh* for upper limbs is similar to the broadband Cuffed Schnogh, but is equipped with several belts in tail portion corresponding to the metal buckles in the head portion.
- Cuffed Schnogh: Each set is a one-piece device designed as twistable by one hand, which is suitable for an arm lymphedema in those who are enthusiastic to do the care without assistant; twisting therefore is to be conducted by the hand of the unaffected side. Of course it is applicable as well to leg/thigh. Each is structured of three portions, a head portion, a body portion, and a tail portion (Fig.1B). The body is shaped like a cuff of the sphygmomanometer (without an inflatable bag) lined externally with loop elements of Velcro tape. The head has a rope work like that of a canvas shoe, underneath of which is lined with an expansible elastic cushion. The ropes are converged onto twisting bars which when twisted can tighten the cuff. The head end supports a buckle for the tail end to lock up the cuff. The tail portion in turn is lined with a hook element of Velcro tape. The body comes as a 15- and 18-cm bandwidth. Normally, for an upper limb two or three, and lower limb four sets of Cuffed Schnogh are required to cover each limb length.
- Multibelted Schnogh: The device was modified and extended after the Cuffed Schnogh for a better fit to the curvature of a swollen arm. Instead of a broad band cut, the tail portion of the cuff is split into a series of 3–9 straps, each 3.8 cm width, so that individual belts can be separately fastened to corresponding buckles (Fig.1C). This type can be custom-made to meet the need of an arm lymphedema patient who wants an all-in-one device; in such a case a full-length, nine-belted Schnogh can be prepared to cover as long as 35 cm along a swollen arm.

- Paired Schnogh: Each set is composed of a male and a female subunit, having a narrowed segment and an elongate perforation, respectively, in the middle part. This comes in three standard sizes: S, 11 × 50 cm; M, 13 × 70 cm; L, 15 × 90 cm. One end is tapered slender, fixed to a metal ring, the other cut full width slightly rounded, attached with a band of either hook or loop element of Velcro tape (Fig.1A). To wrap around a limb, the Velcro-taped ends are coupled and placed over the limb segment. Each free end (with ring) is then inserted under the limb and pulled upward, male going through female perforation, held tight, and both rings paired. A stick is passed into rings and turned around to spirally twist in order to screw up the unused length of the Schnogh. Tightening is then achieved. Depending on the limb length, 6–8 sets are needed to cover a single leg and thigh.

### Schnogh maneuver, a protocol for compression-decompression therapy

The TTT is best practiced in a combination of twist and untwist alternately to generate a compression and decompression effect on the swollen limb. Having tested in preliminary study, we found that 80–90 mmHg is optimum for a 15-min twist followed by a 5-min untwist, that is, three consecutive sessions in 1 h. Treatment of 10 sessions, ∼3½ h a day, is normally acceptable by most individuals. This regimen is the core therapy in reduction phase, which was performed intensively during the ambulatory admission program (one 5-day course, or more); after discharge each patient would be assigned individually with a home program in maintenance phase, relying on physician-prescribed compression materials which can be revised at next follow-up.

**Figure 2:**
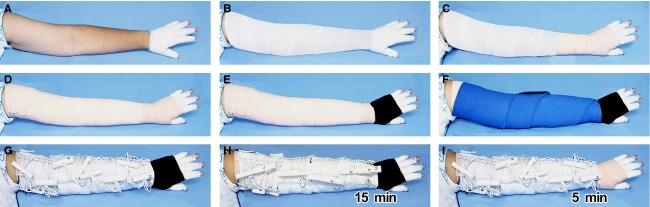
Step-by-step procedures of Twisting Tourquet© Technique demonstrated with Multibelted Schnogh on an upper extremity. (A) Conforming bandage wraps protect all fingers and dorsum of hand. (B) Conforming bandage continues to wrap the entire length of lower and upper arm. (C and D) For a second layer, a long-stretch elastic bandage covers the hand and arm. (E) A third layer in hand is applied with a Velcro-closure elastic wrap (black). (F) A third layer in arm is applied with a neoprene wrap (bright blue). (G) Four units of Multibelted Schnogh are mounted on arm. (H) Twisting bars are kept tightened for 15 min, with male and female bars interlocked. (I) Bars are released for 5 min, Schnogh still kept in place loosely to be twisted again in the next session. Similarly, the Velcro elastic wrap is removed from hand.

**Figure 3:**
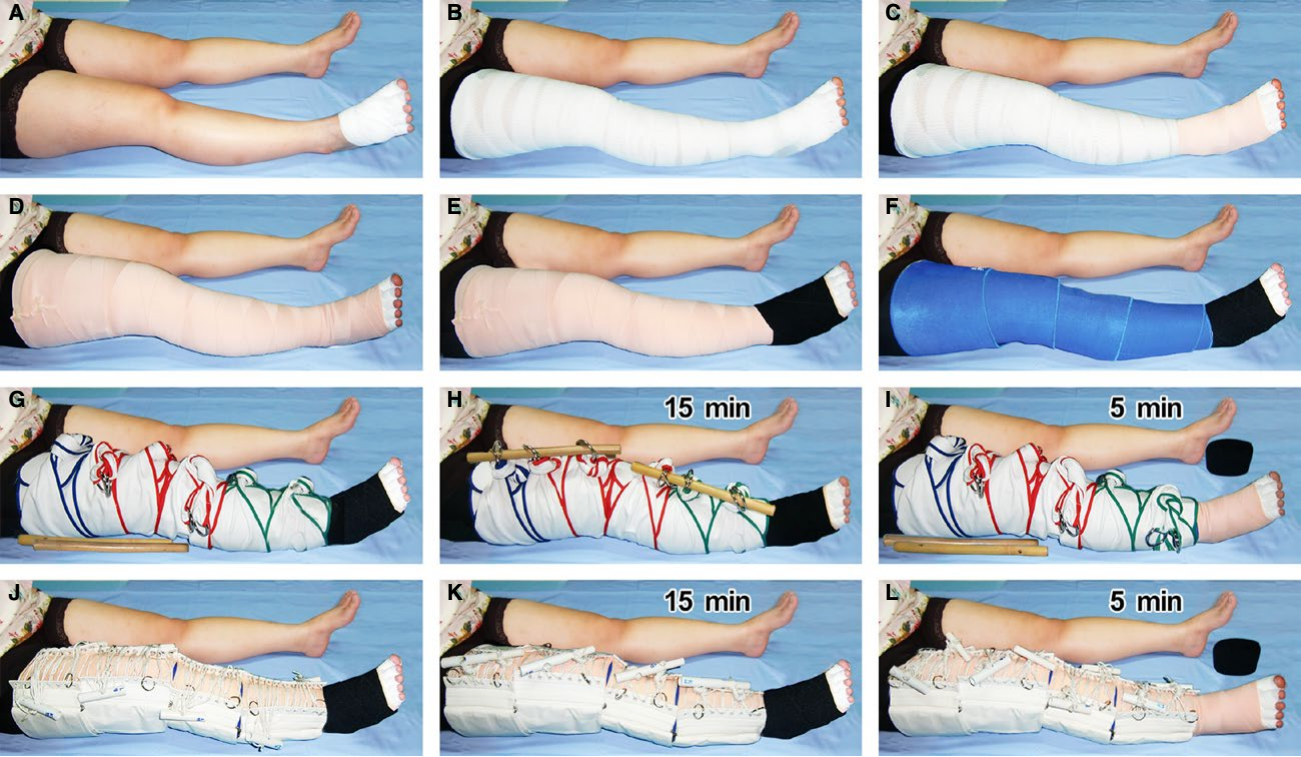
Step-by-step procedures of Twisting Tourquet© Technique demonstrated with Paired Schnogh (A–I) and Cuffed Schnogh (A–F and J–L) on a lower extremity. (A) Conforming bandage wraps protect all toes and dorsum of foot. (B) Conforming bandage continues to wrap the entire length of leg and thigh. (C and D) For a second layer, a long-stretch elastic bandage covers the foot, ankle, leg, and thigh. (E) A third layer in foot is applied with a Velcro-closure elastic wrap (black). (F) A third layer in leg and thigh is applied with a neoprene wrap (bright blue). (G) Six units of Paired Schnogh (green for S, red for M, and blue for L size) are mounted on leg and thigh. (H) Twisting rods are kept tightened for 15 min. (I) Rods are released for 5 min, Schnogh still kept in place loosely to be twisted again in the next session. The Velcro elastic wrap is accordingly removed from foot for 5 min. Alternately, (J) Four units of Cuffed Schnogh are mounted on leg and thigh. (K) Twisting bars are kept tightened for 15 min, male bars inserted into female ones to secure locking. (L) Bars are released for 5 min, Schnogh still kept in place loosely to be twisted again in the next session. The Velcro elastic wrap is temporarily removed from foot.

In actual regimen, a limb is prepared as illustrated in Figures2 and 3. Conforming bandages were used to wrap fingers or toes, with tips opened, hand or foot, and the entire limb length. A second layer was covered by elastic bandages, and a third layer by a neoprene wrap. The fourth layer was for mounting the Schnogh. At the dorsum of hand or foot, instead of Schnogh we applied a *One-Touch Free Supporter* (Daiya, Okayama, Japan), which is a free-wrapping elastic material with Velcro closure. Schnogh pressure at the distal segment was twisted to 80–90 mmHg, and was slightly lessened 3–5 mmHg for each segment to generate a descending gradient distoproximally. Calibration was done by a body-surface pressure gauge (Cape, Tokyo, Japan). In precaution to any adverse reaction that might incur during the procedure, we routinely instruct every patient to not ignore any sign of changes such as pain, numbness, itch, discoloration in finger/toe tips, and any subjective discomfort; such complaints are easily erased by untwisting Schnogh, readjusting position, correcting posture, or unmounting and remounting the Schnogh.

### Patient collections

An ethical clearance was derived from Ethics Committee (Faculty of Tropical Medicine). During 2006 through 2013, more than 3500 patients consulted the Institute for lymphedema, bad lymph sickness, or other poor lymph disorder; they were all examined and diagnosed by a single physician (W. E.). Patients ages ranged from 1 month to 98 years. For this study, inclusion criteria indicated that the individuals had lymphedema in a single limb, either upper or lower extremity, of which diagnosis was confirmed by magnetic resonance imaging (MRI) with T1W and T2W STIR/fs (short T1 inversion recovery with fat suppression modality). Each patient signed an informed consent, enrolled to the program, and completed the 5-day treatment course (5 days within 1 week only and not otherwise). If active underlying diseases were present, the patients were excluded from the analysis. Throughout the course, vegan diet therapy was encouraged to all patients as a means to combat and prevent cellulitis without medication, which was evidently proved effective on follow-up program. With the stated criteria, we could include 647 patients, 307 of upper, and 340 of lower limb group. Demographic data were collected and limb volume assessment calculated.

### Limb volume assessment

**Figure 4:**
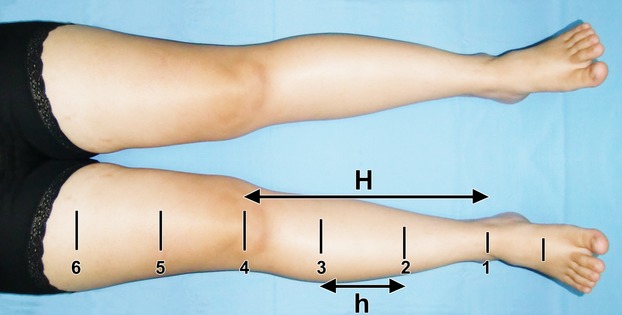
The six sites of circumference measurement in lower limb are determined based on the anatomical landmarks, that is, malleoli (1) and patella (4). The distance between the two is H, one-third of it is an h. The latter's interval length is extended into the thigh subsegments.



Circumference measurement was obtained at seven sites based on anatomic landmarks, that is, midpalm, ulnar styloid, and olecranon in upper limb, and midarch, malleolus, and midpatella in lower limb. Six segments were used for truncated-cone volume approximation 10 to calculate five cones; segment interval length was derived from one-third of lower arm or of leg, such as shown in Figure4.

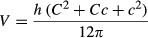
where, *V* = volume of limb at each segment; *C*, *c* = circumference at distal and proximal end, respectively; *h* = *C*-to-*c* interval, or segment length (height).

Based on the calculated volume, before the treatment commenced on day 1, we measured also the unaffected limb to obtain the degree of edema severity, ES (%), and after the course terminated on day 5, we assessed the rate of volume reduction, VR (%), as depicted.

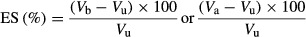


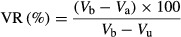
where, *V*_b_ = volume of affected limb before treatment; *V*_a_ = volume of affected limb after treatment; *V*_u_ = volume of unaffected limb.

## Results

### Demographic findings

By the stated inclusion criteria, 647 patients with a single upper limb or lower limb lymphedema were included in the study, of which 570 (88%) were female. Itemized data are depicted in Table1.

**Table 1:** Demographic characteristics of two groups of patients with lymphedema in upper and lower extremity (*n* = 647)

Items	Upper extremity group (*n* = 307)	Lower extremity group (*n* = 340)
Gender		
Female	302 (98.4%)	268 (78.8%)
Male	5 (1.6%)	72 (21.2%)
Age (years)		
Average (range)	58 (12–85)	52 (6–82)
BMI (kg/m^2^)		
Average (range)	26.0 (15.4–51.9)	27.4 (16.1–91.6)
Duration of swelling (months)		
Average (range)	57.0 (0.5–744)	103 (1–684)
Diagnosis		
Primary lymphedema	7 (2.3%)	97 (28.5%)
Secondary lymphedema	300 (97.7%)	243 (71.5%)
Breast cancer	292 (95.1%)	–
Cervical/uterine/ovarian cancer	–	183 (53.8%)
Trauma/surgery	4 (1.3%)	14 (4.1%)
Cellulitis/inflammation	–	8 (2.4%)
Miscellaneous	4 (1.3%)	38 (11.2%)

#### Upper extremity group

Of the 307 patients with unilateral upper limb lymphedema, 302 cases (98.4%) were female. Average age was 58 years. Average body mass index (BMI) was 26.0 kg/m^2^. The duration of swelling was 57 months, the longest one being 62 years of history. Out of these 307 patients, primary lymphedema in arm was found in seven cases (2.3%), whereas 300 (97.7%) were secondary lymphedema, majority, 292 cases (95.1%), of which were related to breast cancer treatment. Arm lymphedema in male was found in five cases (1.6%), two primary and three secondary.

#### Lower extremity group

Of the 340 patients with unilateral lower limb lymphedema, 268 cases (78.8%) were female. At first visit, the age was 52 years, and average BMI 27.4 kg/m^2^. The duration of disease burden was 103 months, the longest one being 57 years of suffering. Of the 340 cases of lower extremity lymphedema, 97 cases (28.5%) were primary and 243 cases (71.5%) were secondary lymphedema, in which most, 183 cases (53.8%), were associated with treatment of cervical cancer and intrapelvic malignancy.

### Volumetric analysis: swelling reduced to half in 5 days

The therapeutic results of TTT in patients with single limb lymphedema who have completed the 5-day treatment course are shown in Table2.

**Table 2:** Therapeutic results of upper and lower extremity lymphedema before and after 5-day Twisting Tourniquet^©^ Technique

Assessment item	Upper extremity group (*n* = 307)Average (range)	Lower extremity group (*n* = 340)Average (range)
Unaffected limb volume, mL	1536 (604–4393)	6031 (1699–17,661)
Affected limb volume before, mL	2516 (948–6802)	9704 (2383–38,983)
Affected limb volume after, mL	2052 (784–5278)	7849 (2068–22,817)
Edema volume before, mL	979 (101–4417)	3674 (241–21,770)
Edema volume after, mL	516 (−14 to 1926)	1818 (−144 to 13,501)
Edema severity before, %	65.4 (6.1–274.7)	61.3 (5.7–368.9)
Edema severity after, %	34.7 (−0.7 to 170.7)	30.6 (−1.1 to 212.7)
Edema volume reduction, mL	463 (65–3019)	1856 (227–16,378)
Rate of volume reduction, %	50.2 (16.5–112.6)	55.6 (17.8–113.5)

#### Upper extremity group

**Figure 5:**
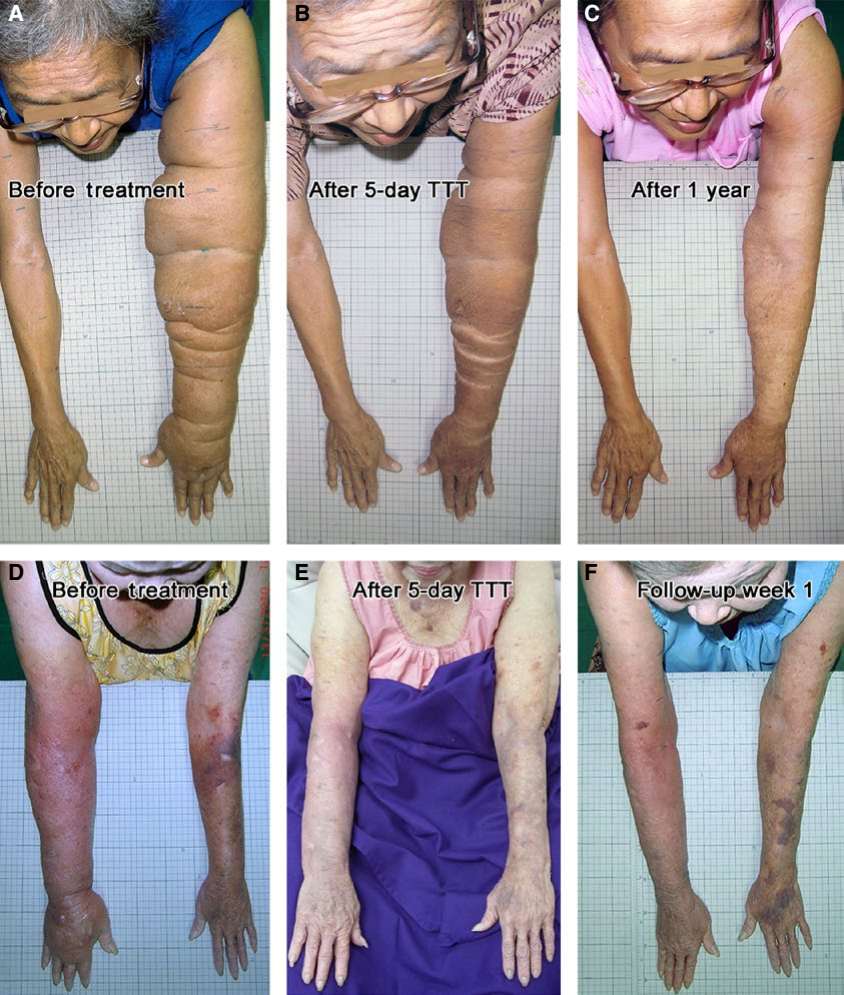
Examples of upper limb cases in secondary (A, B, and C) and primary (D, E, and F) lymphedema. A breast cancer-related lymphedema involved four segments of left arm, fingers, dorsum of hand, forearm, and upper arm, of a 69 y/o female who underwent a standard (mastectomy, chemoradiotherapy, and hormone therapy) treatment for breast cancer 9 years ago, but developed swelling in the last 4 years (A). After a 5-day course with Schnogh compression–decompression therapy, the edema reduction was 66.3% (B). Under successive follow-ups with assigned home program, the arm resumed almost near normalcy (C). Notice also changes in the skin tone and disappearance of forehead folds, as the lymph has been appropriately drained and cleansed by vegan diet. In lower panels (D, E, and F), a woman aged 78 years, without history of breast malignancy, presented a diffuse erythematous swelling in right arm with a few unruptured bleb formations; left arm was slightly swollen but widely laden with petechial macules due to intravenous antibiotic infusion conducted intensively elsewhere (D). Every clinical manifestation was almost cleared after a 5-day Twisting Tourniquet^©^ Technique program (E); note also the fingers and dorsum of hand now becoming slender. However, general erythema resumed and petechial marks recurred a week later, when the patient returned to her fond animal-based diet, especially crabs (F).

Of the 307 patients, the unaffected arm volume was 1536 mL (range 604–4393 mL). The affected arm volume before and after treatment was 2516 and 2052 mL, respectively. The ES decreased from 65.4% to 34.7%, after TTT, with an average edema decrement at 463 mL (range 65–3019 mL), resulting in a rate of VR at 50.2% (range 16.5–112.6%) within 5 days. Examples of secondary and primary lymphedema, before and after TTT, are demonstrated in Figure5.

#### Lower extremity group

**Figure 6:**
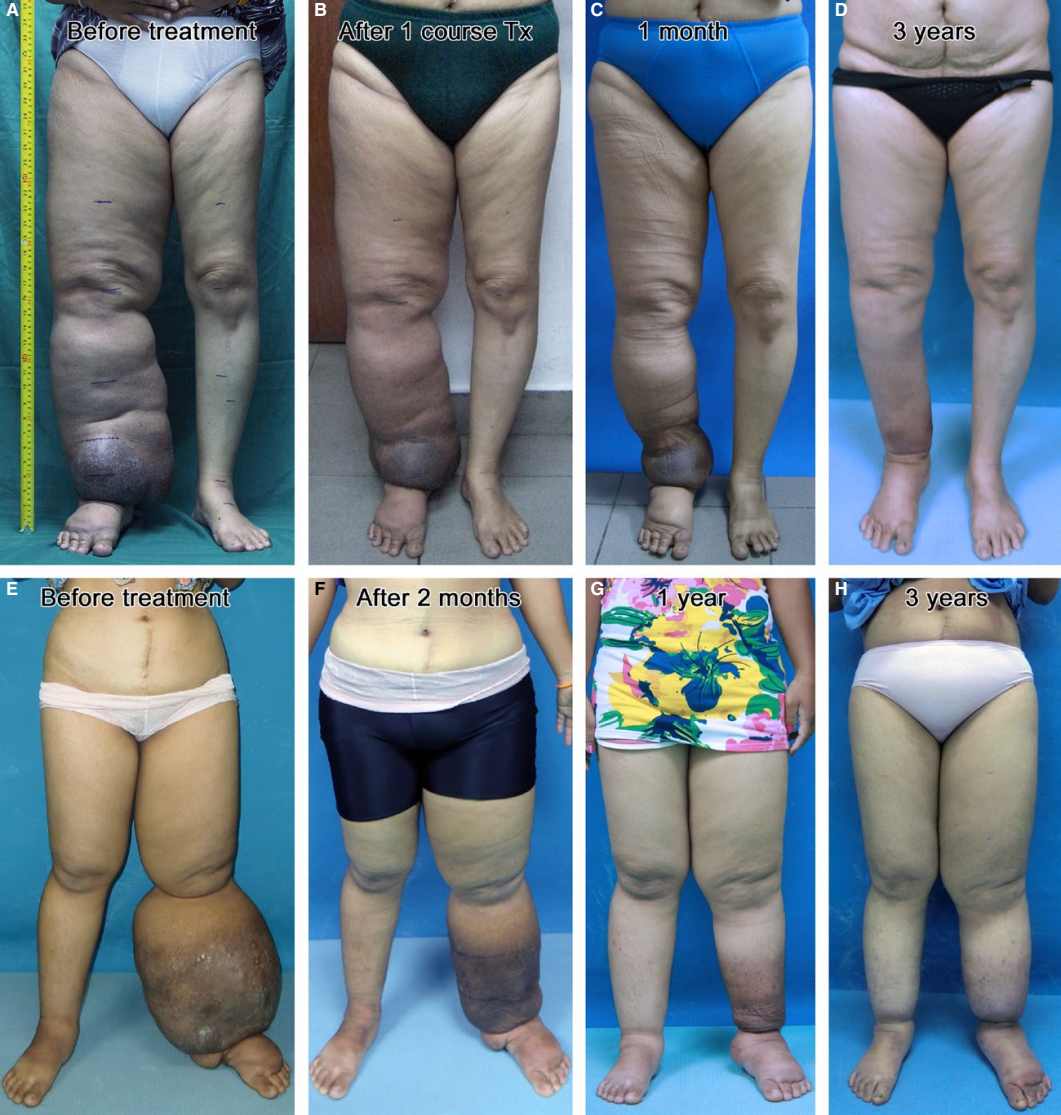
Secondary lymphedema (A) developed in the last 10 years of a mother of three children who had cervical cancer 20 years ago. After a course of TTT (B), she adhered strictly to the treatment plan with an exciting outcome at 1 month (C), and a near-normal cure at 3 years (D). Without any plastic surgery, excessive skin recoiled nicely and hyperpigmentation reduced almost completely. A 30-year-old mother of two children suffered from primary lymphedema (E) since primary school. Having enrolled in the TTT program, she performed excellently at month 2 (F), year 1 (G), and year 3 (H). During year 2, between (G and H), a minor surgery was conducted to remove fat deposit at medial malleolus.

Of the 340 patients, the unaffected lower limb volume was 6031 mL (range 1699–17,661 mL). The affected lower limb volume before and after treatment was 9704 and 7849 mL, respectively. The ES decreased from 61.3% to 30.6% after TTT, with an average edema decrement at 1856 mL (range 227–16,378 mL), resulting in a rate of VR at 55.6% (range 17.8–113.5%) within 5 days. Examples of secondary and primary lymphedema, before and after TTT, are depicted in Figure6.

### Rate of VR varies with class of ES

Due to the large size of patient numbers covering a striking spectrum of severity, we attempted categorizing the individual swelling by grading the ES. The latter was classified into eight grades, mild, moderate, severe (from 1+ to 3+), and gigantic (1+ to 3+); their VR rate appeared as demonstrated in Tables3 and 4 for upper and lower limb group, respectively.

**Table 3:** Edema severity of arm lymphedema is graded into eight classes as each responded with differing volume reduction rate after 5-day Twisting Tourniquet^©^ Technique

Grading		Edema severity%	No. of patients(*n *= 307)	Volume reduction rate% Average ± SD
I	Mild	0–20	29	72.1 ± 22.6
II	Moderate	Up to 40	64	53.5 ± 16.4
III	Severe 1+	Up to 60	67	49.1 ± 13.5
IV	Severe 2+	Up to 80	62	45.6 ± 10.8
V	Severe 3+	Up to 100	31	44.8 ± 8.5
VI	Gigantic 1+	Up to 150	40	41.8 ± 14.1
VII	Gigantic 2+	Up to 200	11	49.5 ± 12.3
VIII	Gigantic 3+	Above 200	3	57.5 ± 17.1

**Table 4:** Edema severity of lower limb lymphedema is graded into eight classes as each responded with differing volume reduction rate after 5-day Twisting Tourniquet^©^ Technique

Grading		Edema severity%	No. of patients(*n* = 340)	Volume reduction rate% Average ± SD
I	Mild	0–20	38	81.7 ± 20.0
II	Moderate	Up to 40	87	60.4 ± 18.2
III	Severe 1+	Up to 60	74	50.9 ± 16.9
IV	Severe 2+	Up to 80	56	50.7 ± 13.9
V	Severe 3+	Up to 100	34	47.9 ± 16.3
VI	Gigantic 1+	Up to 150	39	45.5 ± 11.5
VII	Gigantic 2+	Up to 200	7	48.6 ± 8.2
VIII	Gigantic 3+	Above 200	5	37.1 ± 11.2

## Discussion

There is little doubt that Schnogh under TTT is effective. Schnogh is a progressive tightening device, applied by transforming power of hand-twisting rotation into circumferentially constricting compression force around an axis. In theory an increase in lymph transport, if achievable, should benefit all forms of edema 11. By creating optimally high resting pressure against the tissues and vessels, Schnogh is thought to accelerate venous and lymphatic drainage. The mainstay of TTT relies on the findings that controlled prolonged compression under subsystolic pressure within range tolerable by individuals, alternate with decompression, can induce rapid reduction in swelling without causing detrimental effect. We found that the combination of gradually increasing constriction force by Schnogh until desired pressure, maintained for 15-min, and a following released pressure for at least 5-min, when conducted continually in a repeated compression–decompression manner for 10 sessions a day can generate acceptable results. With an easy-to-use design, TTT is simple so that no technical training is required. The compression–decompression mode of TTT is very powerful and therefore very swift in edema reduction within short days even in the gigantic grade of lymphedema.

In our TTT protocol, we employed no manual lymphatic drainage which is one of the major treatment components that constitute the core element of Complete Decongestive Physiotherapy (CDP or CDT), and is regarded by many as a “gold standard” for lymphedema treatment 12–15. The practice of TTT for upper and lower extremities requires multilayer wrapping with different materials included, Schnogh being the outermost. Skeptics usually fear that TTT might cause ischemia to limbs. Schnogh is different from the surgeons' tourniquets, which operate far above systolic pressure in order to completely block the circulation to result in a bloodless surgical field, generally 250–300 mmHg 16. TTT operates ∼ at 80–90 mmHg pressure in a time limited manner, not more than 15 min, therefore is incapable of causing an arterial occlusion.

Prolonged compression is considered theoretically of threefold benefits:

1. To initiate propulsion of interstitial fluid through honeycomb microchambers 7 toward lymphatic lumens, hence the definite collapse of expanded interstices, shrinkening the thickness of interstitial mass.
2. To inhibit superficial capillary perfusion, hence the temporary suppression of transcapillary hydrostatic fluid movement, limiting the progression of swelling.
3. To decrease vascular space especially of venolymphatic tributaries (low-pressure system), hence the reduction in local intravascular volume, temporarily increasing systemic venous return with urine output.

The alternating decompression interval is of equal importance, more or less analogous to the 10-min “breathing spell” in surgical tourniquet application 17. More similarly, whether high or low pressure, compression bandage results in a better VR if the bandaging is renewed frequently 18.

According to Zuther 15, compression causes a significant shift of fluid into the central parts of the body and thereby increases the central venous blood volume, the heart minute volume, and diuresis. In our experience, during the 5-day course, usually a definite weight reduction with frequent urination was observed.

In the present study, we reported from 307 and 340 patients, after a 5-day TTT, an average VR rate at 50.2% and 55.6% that translate into average edema reduction volume at 463 and 1856 mL, in upper and lower limb, respectively (Table2).

In order to compare results among many different centers, there has been need to unify therapeutic components and shorten days of treatment, which is still difficult to meet. As to the length of duration, in our TTT with Schnogh compression–decompression, we conducted a uniformed 5-day treatment, while the duration could variously be, for instance, 15.7 days (range 4–25 days) using a CDP by Ko and coworkers 19, 6 days (range 3–26 days) for upper limb, and 10 days (range 2–26 days) for lower limb, using a CDP by Yamamoto and Yamamoto 20, 12 days for upper limb 21 and 12.6 days for lower limb 22, using a CDP by Liao and colleagues 21,22, or 8 ± 3 days using an intensive short-term decongestive lymphatic therapy by Szuba and coworkers 23, to state but a few. Interestingly, the so-called CDP employed by these groups 19–22 was never identical, each containing varying proportions of therapeutic components.

Not unlike medical treatment in other disciplines, most lymphedema therapists recognize the dose–response relationship in treating the swelling and tend to perform higher doses (times and days) to obtain better results. Those who reported an above-half improvement, that is, more than 50% VR, extended the treatment for longer days 19–22, but those who shortened days of treatment, 8 ± 3 days 21, their results were notably mixed, 44 ± 62% and 42 ± 40% in upper and lower limb, respectively.

In addition to the duration of treatment, the ES before treatment and the initial volume are also important factors that determine clinical outcome. Ramos and colleagues found that the key to predicting successful lymphedema treatment is the initial volume of edema in the tissues regardless of whether the intervention is early or late 23. Those patients having the lowest volumes of edema fluid have the best chance for a successful outcome. The reason for this may be that those patients with the lowest volume of lymphedema do not have the extensive fibrosis, loss of elasticity, and other pathological changes in the tissues that patients with large volumes normally exhibit, allowing for decongestive therapy to decrease the volume of edema much more effectively.

From our data, Tables3 and 4, it is clear that the lower the ES, the higher rate the reduction response; this holds true for both upper and lower extremity lymphedema. High-grade ES, such as gigantic lymphedema, tended to respond less. In addition, of same severity grading, swelling in lower extremity reacted to treatment more favorably than that of upper extremity.

This study represents the largest population reported, 307 upper limbs and 340 lower limbs, totaled to 647 patients with lymphedema. These large numbers amassed patients with a notably broad range of ES rate of 6.1–274.7% and 5.7–368.9% in upper and lower limb group, respectively (Table2); in which two-thirds are extreme cases graded as severe (ES 40–100%) and gigantic (ES >100%), one-third are mild (ES up to 20%) and moderate (ES up to 40%) as depicted in Tables3 and 4.

In the present study, we attempted classifying severity into Grade I through Grade VIII, based on ES rate (Tables3 and 4); here we call gigantic only when ES rate exceeds 100%, that is, the swollen limb is more than two times by volume.

Patients of advanced grades with monstrous lymphedema who present extreme swelling volume of more than 100% to as much as 400%, can easily employ this technique to reduce the swelling.

Schnogh is an integral element in treating lymphedema during the reduction phase, especially the first week/month, after which it is used only occasionally or null in the maintenance phase, but adopting compression garments or bandaging materials instead.

## Conclusions

From a collection of more than 3500 lymphedema patients, with the stated inclusion criteria, 647 were included in the present study to analyze the therapeutic effectiveness of Schnogh. The latter is a progressive tightening device used under a TTT to manually conduct a compression–decompression maneuver. All patients underwent a uniformed treatment of 3.5 h daily for 5 days. The edema volume reduced in average 50.2% and 55.6% for the upper and lower limb group, respectively. As compared to the severe–gigantic grades, patients of mild–moderate grades of ES responded far more favorably to the TTT.
